# Retraction: Bilateral Compartment Syndrome Affecting the Upper Limbs in a Patient With Chronic Myeloid Leukemia

**DOI:** 10.7759/cureus.r153

**Published:** 2024-12-06

**Authors:** Abhinav Varadhan, Aswinishree Bagavandoss, Christopher T Edmunds, Samson O Oyibo

**Affiliations:** 1 Emergency Medicine, Peterborough City Hospital, Peterborough, GBR; 2 Internal Medicine, Peterborough City Hospital, Peterborough, GBR; 3 Critical Care Medicine, Peterborough City Hospital, Peterborough, GBR; 4 Diabetes and Endocrinology, Peterborough City Hospital, Peterborough, GBR

This article has been retracted by the Editors-in-Chief due to accidental duplicate publication. Due to a communication error among authors, another group from the same institution published the same case report in BMJ Case Reports: https://casereports.bmj.com/content/17/10/e261107.long. As the other article was submitted and published first, this article has been retracted. The authors agree with this decision.

